# Treatment with *α*-Lipoic Acid over 16 Weeks in Type 2 Diabetic Patients with Symptomatic Polyneuropathy Who Responded to Initial 4-Week High-Dose Loading

**DOI:** 10.1155/2015/189857

**Published:** 2015-08-04

**Authors:** Hector Garcia-Alcala, Celia Isabel Santos Vichido, Silverio Islas Macedo, Christelle Nathalie Genestier-Tamborero, Marissa Minutti-Palacios, Omara Hirales Tamez, Carlos García, Dan Ziegler

**Affiliations:** ^1^Departamento de Ciencias de Salud, Facultad de Medicina, Universidad Popular Autónoma del Estado de Puebla, Calle 21 Sur 1103, Barrio de Santiago, 72410 Puebla, Mexico; ^2^Outpatient Diabetes Clinic PEMEX, Calle 46 Poniente 1502, Colonia Cleotilde Torres, 72050 Puebla, Mexico; ^3^Hospital Universitario de Puebla, Benemerita Universidad Autonoma de Puebla, Avenida 25 Poniente 1301, Colonia Los Volcanes, 72410 Puebla, Mexico; ^4^Institute for Clinical Diabetology, German Diabetes Center, Leibniz Center for Diabetes Research at Heinrich Heine University, Auf'm Hennekamp 65, 40225 Düsseldorf, Germany; ^5^Department of Endocrinology and Diabetology, Medical Faculty, Heinrich Heine University, Moorenstraße 5, 40225 Düsseldorf, Germany

## Abstract

Effective treatment of diabetic sensorimotor polyneuropathy remains a challenge. To assess the efficacy and safety of *α*-lipoic acid (ALA) over 20 weeks, we conducted a multicenter randomized withdrawal open-label study, in which 45 patients with type 2 diabetes and symptomatic polyneuropathy were initially treated with ALA (600 mg tid) for 4 weeks (phase 1). Subsequently, responders were randomized to receive ALA (600 mg qd; *n* = 16) or to ALA withdrawal (*n* = 17) for 16 weeks (phase 2). During phase 1, the Total Symptom Score (TSS) decreased from 8.9 ± 1.8 points to 3.46 ± 2.0 points. During phase 2, TSS improved from 3.7 ± 1.9 points to 2.5 ± 2.5 points in the ALA treated group (*p* < 0.05) and remained unchanged in the ALA withdrawal group. The use of analgesic rescue medication was higher in the ALA withdrawal group than ALA treated group (*p* < 0.05). In conclusion, in type 2 diabetic patients with symptomatic polyneuropathy who responded to initial 4-week high-dose (600 mg tid) administration of ALA, subsequent treatment with ALA (600 mg qd) over 16 weeks improved neuropathic symptoms, whereas ALA withdrawal was associated with a higher use of rescue analgesic drugs. This trial is registered with ClinicalTrials.gov Identifier: NCT02439879.

## 1. Introduction

Type 2 diabetes mellitus is one of the most prevalent disorders in Mexico and worldwide. Based on the results of the Mexican Health and Nutrition Survey 2012 (Encuesta Nacional de Salud y Nutrición; ENSANUT 2012), there are 6.4 million diagnosed diabetic individuals in Mexico, and 1.8 million have diabetic complications [1]. Since the chronic diabetic complications are associated with considerable morbidity and increased mortality and have a direct impact on health care costs, it is important to seek for effective treatments that reduce some of the burden associated with chronic diabetic microvascular complications such as neuropathy [2].

DSPN is encountered in about one-third of all diabetic patients [3] and predicts cardiovascular morbidity [4] and mortality [5]. Painful neuropathy is observed in 13–26% of individuals with diabetes and exerts a substantial impact on the quality of life [3]. Intensive diabetes therapy is considered the causal approach to prevent and treat DSPN, but current evidence suggests that optimized glycemic control usually is insufficient to fully prevent the development and progression of DSPN, especially in type 2 diabetic patients [6]. Moreover, near-normoglycemia is difficult to achieve in a considerable number of individuals with diabetes. Symptomatic treatment of neuropathic pain using analgesic monotherapy is generally only modestly effective [7, 8], and inadequate response to drug treatments constitutes a substantial unmet need in patients with neuropathic pain [7]. Moreover, these drugs have been designed solely to relieve pain, but not to favorably influence the pathophysiology of the underlying neuropathy.

Oxidative stress plays a major role in the pathogenesis of diabetic microvascular complications including neuropathy [9, 10]. Based on the putative mechanisms underlying DSPN, several therapeutic approaches have been developed including antioxidants such as *α*-lipoic acid (ALA) to diminish enhanced oxidative stress [11, 12]. In the NATHAN 1 study, 4-year treatment with ALA in mild-to-moderate essentially asymptomatic DSPN resulted in a clinically meaningful improvement and prevention of progression of neuropathic impairments [13]. We previously reported in a meta-analysis including the 3-week trials that treatment with ALA (600 mg/day i.v.) improved both positive neuropathic symptoms and neuropathic deficits to a clinically meaningful degree in diabetic patients with symptomatic DSPN [14]. Two recently published meta-analyses [15, 16] confirmed these findings. However, it was concluded that when given i.v. at a dosage of 600 mg/day over a period of 3 weeks, ALA leads to a clinically relevant reduction in neuropathic pain, but it remains unclear if the significant improvements seen after 3–5 weeks of oral administration [17, 18] are clinically relevant [16]. Thus, further studies in symptomatic DSPN using oral ALA over longer time periods are warranted.

The objective of this multicenter enriched enrolment randomized withdrawal open-label study was to assess the efficacy and safety of ALA using 600 mg qd over 16 weeks in patients with type 2 diabetes and symptomatic DSPN who responded to 4-week antecedent initial treatment with 600 mg tid.

## 2. Materials and Methods

This trial was conducted in accordance with the Declaration of Helsinki and was approved by the ethics committee of Universidad Popular Autónoma del Estado de Puebla, Mexico. All participants provided a written informed consent (ClinicalTrials.gov Identifier: NCT02439879). Type 2 diabetic patients (according to American Diabetes Association (ADA) criteria) with symptomatic DSPN defined as the presence of neuropathic symptoms (pain, paresthesias, or numbness) were invited to participate in this open-label multicenter enriched enrolment randomized withdrawal trial. Inclusion criteria were a Total Symptom Score (TSS) [14] >7 points, HbA1c < 10%, and serum creatinine <2 mg/dL. Exclusion criteria were evidence of active cardiovascular disease, malignancy, or any other conditions causing neuropathic pain, use of analgesic, antidepressant, or antiepileptic drugs, or any other medication aimed at reliefing neuropathic pain. In addition, child-bearing female patients not using any effective birth control method and under surveillance of a board-certified gynecologist were excluded.

*Phase 1*. All patients meeting inclusion criteria received 600 mg of *α*-lipoic acid (ALA) (Meda Pharma, Germany) orally tid, 30 min after each main meal for 4 weeks. During phase 1, no medication for relief of neuropathic pain was allowed. Each participating site was in charge to maintain glycemic control based on the investigator's judgment attempting that all patients were treated according to the ADA guidelines. All patients were seen once a week, and, at each site visit, TSS was assessed along with a pill count to ensure drug adherence, presence of adverse events, and, if needed, treatment adjustments to maintain glucose levels within the ADA targets. Patients with a TSS reduction >3 points by the end of phase 1 were selected to proceed with phase 2 of the study. Patients with a decrease <3 points in TSS or who used other neuropathic pain drugs were excluded from study phase 2.

*Phase 2*. Patients with a decrease of ≥3 TSS points after phase 1 were randomized to receive 600 mg of ALA orally qd for 16 weeks or ALA withdrawal. Patients were scheduled to visit the clinic every 2-3 weeks for TSS, monofilament, and assessment. If needed, the patient was prescribed analgesic rescue medication which was monitored at each visit. Primary endpoint was the change in TSS consisting of four individual components (burning pain, lancinating pain, paresthesias, and numbness) in the two groups studied in phase 2.

Neurological examination was performed at baseline and after phases 1 and 2 including the monofilament test, vibration perception threshold (VPT), and ankle reflexes. A 10 g nylon monofilament (Thio-Feel Meda Pharma, Germany) was applied to four anatomical sites in each foot (1st, 3rd, and 5th metatarsal heads and plantar surface of distal hallux) as previously described (correct answer = 1 point, with a maximum of 4 points in each foot). Eight correct answers were considered normal and 1–7 correct answers indicated reduced monofilament sensation, while absent sensation was assumed if no answer was correct. VPT was evaluated using a 128-Hz tuning fork (Thio-Vib, Meda Pharma, Germany) applied bilaterally at the tip of the great toe. Responses were categorized as abnormal (no perception of vibration), present (examiner perceives vibration <10 sec after patient reported disappearance of vibration perception), and reduced (examiner perceives vibration >10 sec after patient reported disappearance of vibration perception). Ankle reflexes were graded as normal, decreased, and absent [19, 20].

### 2.1. Statistical Analysis

**Figure 1:**
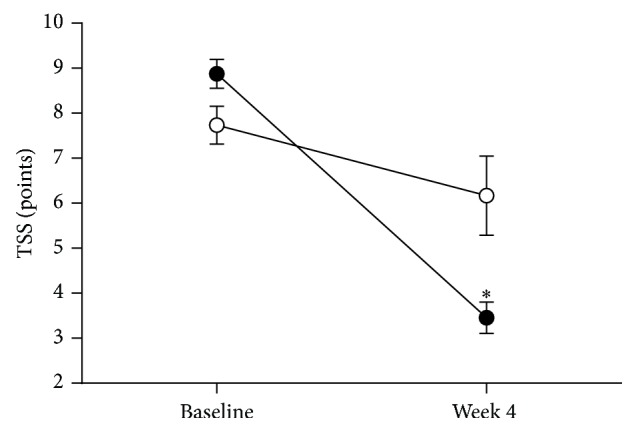
Total Symptom Score (TSS) during the first 4 weeks of treatment with *α*-lipoic acid (600 mg tid) in responders (*n* = 33, solid circles) and nonresponders (*n* = 4, open circles). Values are mean ± SEM; ^*∗*^ *p* < 0.05 versus baseline.

**Figure 2:**
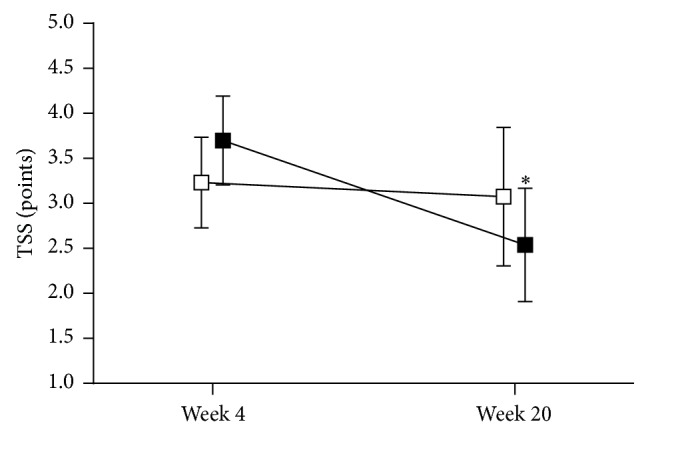
Total Symptom Score (TSS) from 4 to 20 weeks in patients who received *α*-lipoic acid (600 mg qd; *n* = 16, solid squares) compared to those in whom *α*-lipoic acid was withdrawn (*n* = 17, open squares). Values are mean ± SEM; ^*∗*^ *p* < 0.05 versus week 4.

All data were analyzed using the SPSS v16 statistical package software. To compare categorical variables, the *χ* ^2^ test was used, and, for continuous variables, the *t*-test for independent or paired samples was applied. Data are expressed as percent values with 95% confidence intervals (CI) or mean ± SD (Table 1) or mean ± SEM (Figures 1 and 2).

## 3. Results and Discussion

### 3.1. Results

Forty-five patients participated in phase 1 of the study, 12 of whom were excluded due to different reasons including 4 participants with a reduction in TSS < 3 points, 6 subjects who withdrew from the study because of personal reasons, and 2 individuals who used prohibited medication. The demographic and clinical data of the patients at the time of randomization are shown in Table 1. No significant differences between the groups were noted for any of the parameters listed, except for abnormal VPT on the right hallux which was more frequent in the ALA withdrawal group (*p* < 0.05).

**Figure 3:**
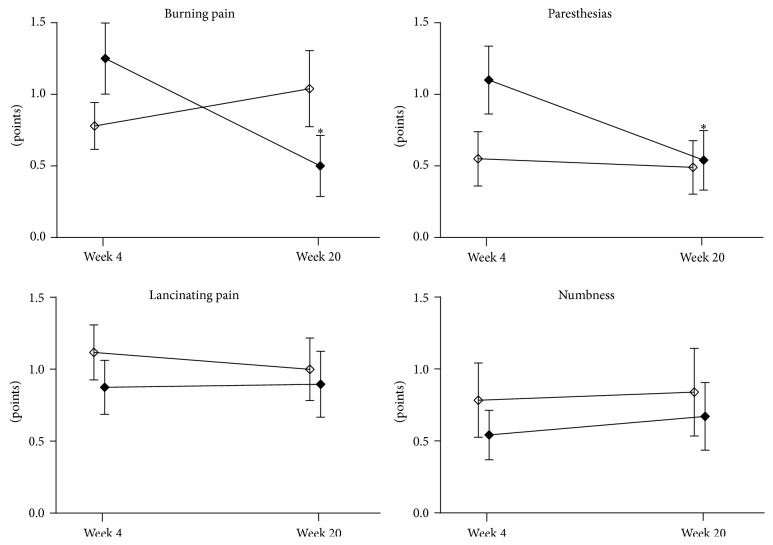
Individual components of the Total Symptom Score (TSS) from 4 to 20 weeks in patients who received *α*-lipoic acid (600 mg qd; *n* = 16, solid squares) compared to those in whom *α*-lipoic acid was withdrawn (*n* = 17, open squares). Values are mean ± SEM; ^*∗*^ *p* < 0.05 versus week 4.

During phase 1, TSS decreased from 8.9 ± 0.3 points to 3.5 ± 0.3 points (*p* < 0.05) in the responder group and from 7.7 ± 0.4 to 6.2 ± 0.9 points in the nonresponders (Figure 1). The course of TSS in the two groups studied during phase 2 is shown in Figure 2. TSS declined from 3.7 ± 0.5 points to 2.5 ± 0.6 points in the ALA treated group (*p* < 0.05), while, in the ALA withdrawal group, TSS remained unchanged with 3.2 ± 0.5 points at randomization and 3.1 ± 0.8 points at study end (*p* = 0.81). The course of the four individual TSS components during phase 2 is illustrated in Figure 3. Burning pain and paresthesias declined from the randomization time to study end (both *p* < 0.05), whereas lancinating pain and numbness remained unchanged in the ALA treated group.

The percentages with 95% CI of patients with reduced sensation to monofilament and vibration and decreased/absent ankle reflex at baseline and after 4 weeks (phase 1) are given in Table 2. VPT on both right and left hallux improved significantly from baseline to 4 weeks, while no significant changes were noted for the remaining neuropathic signs. The percentages with 95% CI of patients with reduced sensation to monofilament and vibration and decreased/absent ankle reflex in the two groups studied during phase 2 are shown in Table 3. No significant differences between the groups were noted for the changes in neuropathic deficits during phase 2.

**Figure 4:**
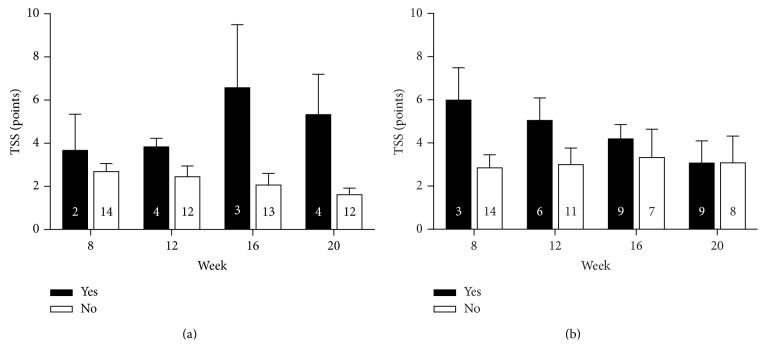
Total Symptom Score (TSS) at weeks 8–20 in participants who received *α*-lipoic acid (a) and those in whom *α*-lipoic acid was withdrawn (b) separately for subgroups which received analgesic rescue medication (black bars) and those who did not (white bars). Numbers in bars represent *n*. Values are mean ± SEM.

The percentages with 95% CI of patients who required analgesic rescue medication during phase 2 are given in 4-week intervals in Table 4. Use of analgesic rescue medication was higher in the ALA withdrawal group than in the ALA treated group (76.5% versus 43.8%, *p* < 0.05). The individual analgesics used as rescue medication are listed Table 5. The course of TSS at weeks 8–20 in participants who received ALA treatment and those in whom ALA was withdrawn separately for the subgroups that received analgesic rescue medication and those who did not is presented in Figure 4. Overall, TSS tended to increase in subjects who received rescue medication in the ALA treatment group, while TSS declined by approximately 50% in the ALA withdrawal group to a level identical to that seen in the subgroup without rescue medication. In subjects not receiving analgesic rescue medication TSS tended to decrease in the ALA treatment group, while it remained fairly constant in the ALA withdrawal group. No significant differences were noted between and within the subgroups that received analgesic rescue medication and those who did not.

In the ALA treated group, HbA1c was 9.3 ± 3.0% at 4 weeks and 8.2 ± 2.2% at study end (*p* = 0.38), while in the ALA withdrawal group HbA1c was 8.1 ± 1.4% at 4 weeks and 7.7 ± 1.7% at study end (*p* = 0.378). No treatment emergent adverse events were observed throughout the study.

### 3.2. Discussion

Pain associated with DSPN exerts a substantial impact on quality of life, particularly by causing considerable interference in sleep and enjoyment of life [21]. However, the impact of DSPN is still being underestimated by both physicians and patients. In one UK survey, only 65% of patients with diabetes received treatment for their neuropathic pain, although 96% had reported the pain to their physician [22]. Another study found that 20% of diabetic patients aged >65 years receiving pain related medications who had diagnosis of peripheral neuropathy within 30 days of such prescriptions were prescribed tricyclic antidepressants (TCAs). Among these patients, nearly 50% had comorbidities and/or received other medications that could render the prescribing of TCAs potentially inappropriate. Thus, many older diabetic patients with DSPN who receive TCA therapy may be inappropriately treated. Safer agents such as ALA may be more appropriate particularly in the older diabetic population [23]. In a German population based survey, 77% of the cases with DSPN were unaware of having the disorder, defined as answering “no” to the question “Has a physician ever told you that you are suffering from nerve damage, neuropathy, polyneuropathy, or diabetic foot?” Approximately one-quarter of the subjects with known diabetes had never undergone a foot examination. Even among individuals with known diabetes who reported to have had their feet examined by a physician, 72% of those with DSPN emerged to be unaware of having DSPN [24]. Thus, there is still a high prevalence of unawareness of having clinical DSPN among diabetic patients and an insufficient frequency of professional foot examinations, suggesting inadequate attention to diabetic foot prevention practice [24]. Likewise, in Mexico, it usually takes a relatively long time before patients with DSPN receive a correct diagnosis and appropriate treatment. In our own survey, we found that diabetic patients suffer from neuropathic pain for about 2 years before they receive professional treatment [25].

Against this background, the results of this multicenter enriched enrolment randomized withdrawal (EERW) open-label study demonstrate that, in type 2 diabetic patients with symptomatic polyneuropathy who responded to initial 4-week high-dose (600 mg tid) administration of ALA, subsequent treatment with ALA (600 mg qd) over 16 weeks resulted in improvement of the TSS. Notably, burning pain and paresthesias rather than lancinating pain and numbness contributed to the TSS improvement in the ALA treated group at study end. In contrast, neuropathic symptoms did not change during withdrawal of ALA for 16 weeks, but this could obviously be accomplished only at the cost of increased use of analgesic rescue medication at week 20 in 53% of the subjects in whom ALA was withdrawn versus 25% of those treated with ALA. Indeed, in subjects receiving rescue medication the TSS declined by approximately 50% in the ALA withdrawal group to the same level as observed in the ALA withdrawal subgroup without rescue medication at study end. In the context of drug mechanism of action it is tempting to speculate that the initial effect on neuropathic symptoms and vibration sensation induced by high-dose ALA treatment was mediated by reduced oxidative stress and may persist for some time. Thus, the finding that efficacy persisted in subjects not taking rescue medication deserves further study. Since the TSS response rates to oral ALA treatment are typically around 50–60% [18], it is not surprising that 4 patients (25%) on ALA required analgesic rescue medication during phase 2. Even in trials using effective analgesics for painful diabetic neuropathy, the mean average daily dose for concomitant use of acetaminophen tends to be similar during placebo treatment (203 mg) and active treatment (152 mg) [26].

The ORPIL [17] and SYDNEY 2 [18] trials previously reported that oral treatment with ALA results in a significant reduction of neuropathic symptoms scored by the TSS in diabetic patients with symptomatic DSPN. However, the duration of ALA treatment in these trials was only 3 and 5 weeks, respectively. Thus, the present study extends the current knowledge by showing that treatment with ALA using a loading dose of 600 mg tid for 4 weeks, followed by 600 mg qd for 16 weeks, is effective in reducing neuropathic symptoms in patients with symptomatic DSPN. We used an EERW trial design to increase sensitivity by removing definite nonresponders and to obtain an indication of overall proportion of patients who benefit [27]. Enriched designs can limit randomization to subjects who have responded to a treatment and/or tolerated its side effects, failed to respond to one or more other active treatments or placebo, or are characterized by various clinical features [27]. Such a design may provide greater assay sensitivity when randomization is limited to subjects who have demonstrated a clinically meaningful treatment response during an open-label phase, and the responses to treatment in the open-label phase are more directly applicable to clinical practice [28]. In fact, an EERW trial design has recently been successfully applied in pivotal studies assessing the efficacy of opioid treatment in painful diabetic neuropathy [29].

During the initial 4-week phase, the TSS was reduced in the responders by almost two-thirds from 8.9 ± 1.8 points to 3.5 ± 2.0 points (*p* < 0.05). Nonetheless, continuation of ALA treatment using 600 mg qd for another 16 weeks resulted in a further TSS decline by 32% and was safe. Given that the circumstance that the TSS level reached in the responders after the initial 4-week phase was already relatively low, we suggest that the mean TSS reduction by 1.2 points after further 16 weeks of ALA treatment is clinically meaningful. This notion is supported by the meta-analysis published by Mijnhout et al. [16], showing a standardized mean difference (95% CI) in TSS between ALA and placebo of 1.78 (1.10–2.45) points. However, it must be kept in mind that the baseline TSS level in the group treated with ALA 600 mg qd in the SYDNEY 2 study [18] was 9.4 ± 1.9 points, similar to the baseline TSS level seen in the present study, but herein the room for further improvement after 4 weeks was much smaller than it was in the SYDNEY 2 study.

One limitation of this study is the open-label study design including a control arm without treatment. Thus, bias due to not including placebo treatment during phase 2 of the study cannot be excluded. Another limitation is that we did not include nerve conduction studies as an objective measure of nerve function. However, the NATHAN 1 study showed that improvement of neuropathic impairments rather than nerve conduction may be expected after 4 years of treatment with ALA [13]. A strength of the present trial is its enriched enrolment design, allowing for the selection of responders, which mirrors the real world approach to treatment more closely than the standard randomized clinical trial.

## 4. Conclusions

In this multicenter enriched enrolment randomized withdrawal open-label study we demonstrated that in responders to initial 4-week high-dose (600 mg tid) administration of ALA, subsequent treatment with ALA (600 mg qd) over 16 weeks effectively diminished neuropathic symptoms, whereas ALA withdrawal was associated with a higher use of rescue analgesic drugs in type 2 diabetic patients with symptomatic DSPN.

## Figures and Tables

**Table 1:** Demographic and clinical data of the patients randomized at week 4 to treatment with *α*-lipoic acid (600 mg qd) or withdrawal of *α*-lipoic acid for 16 weeks (Phase 2).

	*α*-Lipoic acid treatment	*α*-Lipoic acid withdrawal	*p* value
*n*	16	17	—
Sex (% male)	31.3 (13.2–54.8)	35.3 (16.6–58.0)	0.549
Age (years)	57.5 ± 10	59.0 ± 11	0.620
BMI (kg/m²)	27.1 ± 3.6	26.9 ± 4.4	0.898
Systolic blood pressure (mm Hg)	121 ± 10	121 ± 9	0.902
Diastolic blood pressure (mm Hg)	72 ± 9	76 ± 9	0.265
Heart rate (bpm)	76 ± 7	77 ± 7	0.540
Diabetes duration (years)	10.4 ± 7.8	13 ± 5.0	0.259
HbA1c (%)	9.3 ± 3.0	8.1 ± 1.4	0.150
Serum creatinine (mg/dL)	0.82 ± 0.19	0.86 ± 0.23	0.620
OADs (%)	56.3 (33.3–77.3)	41.2 (21.2–63.6)	0.303
OADs and insulin (%)	43.8 (22.7–66.7)	58.8 (36.4–78.8)	0.303
Retinopathy with prior photocoagulation (%)	6.3 (0.3–26.4)	5.9 (0.3–25.0)	0.742
Nephropathy (%)	12.5 (2.3–34.4)	11.8 (2.1–32.6)	0.676
Hypertension (%)	37.5 (17.8–60.9)	52.9 (31.1–74.0)	0.295
Hyperlipidemia (%)	25.0 (9.0–48.4)	29.4 (12.4–52.2)	0.543
Reduced sensation to monofilament (%)	62.5 (39.1–82.2)	58.8 (36.4–78.8)	0.812
Abnormal VPT on right hallux (%)	18.8 (5.3–41.7)	52.9 (31.1–74.0)	0.046
Abnormal VPT on left hallux (%)	31.3 (13.2–54.8)	47.1 (26.0–68.9)	0.284
Decreased/absent ankle reflex right (%)	43.8 (22.7–66.7)	52.9 (31.1–74.0)	0.429
Decreased/absent ankle reflex left (%)	62.5 (39.1–82.2)	58.8 (36.4–78.8)	0.556

Values are mean ± SD or percentages with 95% CI.

OADs: oral antidiabetic drugs; VPT: vibration perception threshold.

**Table 2:** Percentages (95% CI) of patients with reduced sensation to monofilament and vibration and decreased/absent ankle reflex at baseline and after 4 weeks (Phase 1).

	Baseline	4 weeks	*p* value
	% (95% CI)	% (95% CI)	
Monofilament	45.5 (30.5–61.1)	60.6 (44.8–74.9)	0.21
VPT hallux right	66.7 (50.9–80.1)	36.4 (22.5–52.2)	0.01
VPT hallux left	63.6 (47.8–77.5)	39.4 (25.1–55.2)	0.05
Ankle reflex right	69.7 (54.0–82.5)	48.5 (33.3–63.9)	0.07
Ankle reflex left	69.7 (54.0–82.5)	60.6 (44.8–74.9)	0.43

VPT: vibration perception threshold.

**Table 3:** Percentages (95% CI) of patients with reduced sensation to monofilament and vibration and decreased/absent ankle reflex at 4 weeks and study end (Phase 2).

	*α*-Lipoic acid treatment	*α*-Lipoic acid withdrawal	*p* value
	% (95% CI)	% (95% CI)	
Monofilament			
Randomization	62.5% (39.1–82.2)	58.8% (36.4–78.8)	*p* = 0.82
Study end	68.8% (45.2–86.8)	64.7% (42.0–83.4)	*p* = 0.80
*p* value	*p* = 0.70	*p* = 0.72	
VPT hallux right			
Randomization	18.8% (5.3–41.7)	52.9% (31.1–74.0)	*p* = 0.07
Study end	18.8% (5.3–41.7)	35.3% (16.6–58.0)	*p* = 0.43
*p* value	*p* = 1.0	*p* = 0.30	
VPT hallux left			
Randomization	31.3% (13.2–54.8)	47.1% (26.0–68.9)	*p* = 0.35
Study end	25.0% (9.0–48.4)	35.3% (16.6–58.0)	*p* = 0.69
*p* value	*p* = 0.69	*p* = 0.48	
Ankle reflex right			
Randomization	43.8% (22.7–66.7)	52.9% (31.1–74.0)	*p* = 0.59
Study end	25.0% (9.0–48.4)	52.9% (31.1–74.0)	*p* = 0.1
*p* value	*p* = 0.26	*p* = 1.0	
Ankle reflex left			
Randomization	62.5% (39.1–82.2)	58.8% (36.4–78.8)	*p* = 0.82
Study end	37.5% (17.8–60.9)	41.2 (21.2–63.6)	*p* = 0.82
*p* value	*p* = 0.15	*p* = 0.30	

VPT: vibration perception threshold.

**Table 4:** Percentages (95% CI) of patients who required analgesic rescue medication.

Week from randomization	*α*-Lipoic acid treatment	*α*-Lipoic acid withdrawal	*p* value
	% (95% CI)	% (95% CI)	
Week 4	12.5 (2.3–34.4)	17.6 (5.0–39.6)	NS
Week 8	25.0 (9.0–48.4)	35.3 (16.6–58.0)	NS
Week 12	18.8 (5.3–41.7)	52.9 (31.1–74.0)	*p* = 0.04
Week 16	25.0 (9.0–48.4)	52.9 (31.1–74.0)	*p* = 0.09

**Table 5:** Individual analgesics used as rescue medication with number of patients given in brackets.

Week from randomization	*α*-Lipoic acid treatment	*α*-Lipoic acid withdrawal
Week 4	(i) Ibuprofen (1) (ii) Ketorolac (1)	(i) Amitriptyline (1) (ii) Ketorolac (2)

Week 8	(i) Amitriptyline (1) (ii) Ibuprofen (1) (iii) Ketorolac (2)	(i) Amitriptyline (1) (ii) Tramadol (1) (iii) Dextropropoxyphene (1) (iv) Gabapentin (2) (v) Diclofenac (1)

Week 12	(i) Tramadol (1) (ii) Dextropropoxyphene (1) (iii) Ibuprofen (1)	(i) Amitriptyline (2) (ii) Diclofenac plus vitamin B (2) (iii) Meloxicam (2) (iv) Gabapentin (2)

Week 16	(i) Tramadol (1) (ii) Naproxen (1) (iii) Paracetamol (1) (iv) Ibuprofen (1)	(i) Amitriptyline (2) (ii) Diclofenac plus vitamin B (2) (iii) Meloxicam (2) (iv) Gabapentin (2)
